# Intra-Individual Variability in Alzheimer's Disease and Cognitive Aging: Definitions, Context, and Effect Sizes

**DOI:** 10.1371/journal.pone.0016973

**Published:** 2011-04-19

**Authors:** Rochelle E. Tractenberg, Robert H. Pietrzak

**Affiliations:** 1 Departments of Neurology, Biostatistics, Bioinformatics & Biomathematics, and Psychiatry, Georgetown University School of Medicine, Washington, D.C., United States of America; 2 Clinical Neurosciences Division, National Center for Posttraumatic Stress Disorder, VA Connecticut Healthcare System and Department of Psychiatry, Yale University School of Medicine, New Haven, Connecticut, United States of America; 3 Collaborative for Research on Outcomes and Metrics, Washington, D.C., United States of America; McGill University/Douglas Mental Health University Institute, Canada

## Abstract

**Background/Aims:**

To explore different definitions of intra-individual variability (IIV) to summarize performance on commonly utilized cognitive tests (Mini Mental State Exam; Clock Drawing Test); compare them and their potential to differentiate clinically-defined populations; and to examine their utility in predicting clinical change in individuals from the Alzheimer's Disease Neuroimaging Initiative (ADNI).

**Methods:**

Sample statistics were computed from ADNI cohorts with no cognitive diagnosis, a diagnosis of mild cognitive impairment (MCI), and a diagnosis of possible or probable Alzheimer's disease (AD). Nine different definitions of IIV were computed for each sample, and standardized effect sizes (Cohen's *d*) were computed for each of these definitions in 500 simulated replicates using scores on the Mini Mental State Exam and Clock Drawing Test. IIV was computed based on test items separately (‘within test’ IIV) and the two tests together (‘across test’ IIV). The best performing definition was then used to compute IIV for a third test, the Alzheimer's Disease Assessment Scale-Cognitive, and the simulations and effect sizes were again computed. All effect size estimates based on simulated data were compared to those computed based on the total scores in the observed data. Association between total score and IIV summaries of the tests and the Clinician's Dementia Rating were estimated to test the utility of IIV in predicting clinically meaningful changes in the cohorts over 12- and 24-month intervals.

**Results:**

ES estimates differed substantially depending on the definition of IIV and the test(s) on which IIV was based. IIV (coefficient of variation) summaries of MMSE and Clock-Drawing performed similarly to their total scores, the ADAS total performed better than its IIV summary.

**Conclusion:**

IIV can be computed within (items) or across (totals) items on commonly-utilized cognitive tests, and may provide a useful additional summary measure of neuropsychological test performance.

## Introduction

Intra individual variability (IIV) is an important, although underappreciated, aspect of cognitive testing and assessment in elderly individuals who are either at risk for dementia or who have a diagnosis and whose progress is being monitored via cognitive tests [1], [2]. IIV may be overlooked in neuropsychological research and practice because estimates of IIV are almost always based on reaction time and accuracy-based measures (e.g., [3]). Further, there are multiple methods of defining IIV [2], [4]–[6]; without a single best way to compute IIV, it is challenging to introduce –or use - as a summary outside of its most commonly-used context.

While total scores provide an estimate of overall performance on cognitive measures, IIV measures can complement these scores and may improve prediction of global decline [7], functional decline [8], and incident dementia [9]. It has been suggested that estimates of IIV provide a quantitative measure of neurobiological integrity in cognitive aging and neurodegenerative disease [4], [10]–[13], as greater mean IIV levels have been reported in samples with mild cognitive impairment [2] and mild dementia [1], and has been found to associate with decreased frontal gray matter [14], white matter alterations [15], and altered dopaminergic and acetylcholinergic neurotransmission [11]–[12], [16]–[17].

Researchers have been estimating, and interpreting, different patient profiles in IIV with respect to reaction times and “accuracy” (i.e., right/wrong response summary) for at least a decade (see [1], [11]–[12], [18]–[19]). By contrast, cross-domain versions of IIV have also recently been used to estimate IIV using neuropsychological tests (e.g., [7]–[9]). These IIV estimates have all been based on a single formulation of IIV: within-subject standard deviations across cognitive domains –subscales of one test or tests within a battery. Results of these studies have shown that performance on specific subscales of global cognitive tests, instead of the overall score on the test, predicts cognitive change in preclinical Alzheimer's disease [20], and that cross-domain IIV (within-subject standard deviation across subscale) summarizing the test at baseline, predicts cognitive decline over an 18-month period above and beyond mean score performance [7]. However, separable factors or domains on tests such as the Mini-Mental State exam (MMSE [21]) have not been reliably observed [22]–[24], suggesting that IIV estimates based on subscores of the MMSE might not be replicable.

Using the within-individual standard deviation (ISD) definition of IIV, Rapp et al. [8] reported that cross-domain IIV, computed from a battery of neuropsychological tests (i.e., task dispersion [4], [17]) predicted functional decline in both nursing home residents and community-dwelling older adults. Similarly, Holtzer et al. [9] found that across-test IIV predicted incident dementia independent of mean level performance in a population-based study. Hilborn et al. [6] also studied dispersion of performance across tasks and found that this definition of IIV was significantly associated with the likelihood of decline from estimated prior IQ, particularly older old (75–92 years old), as well as with poorer health and demographic characteristics. Using another definition of IIV, Duchek et al. [25] found that within-test IIV (coefficient of variation, not within-subject standard deviation), derived from attention tasks, was associated with a genetic marker (ApoE) and with cerebrospinal fluid biomarkers believed to be associated with Alzheimer's disease.

Focusing on items within a single cognitive test, Tractenberg, Yumoto et al. [26] found that levels of IIV in item-level performance on a commonly utilized measure of “global” cognitive function (Mini Mental State Exam (MMSE), [21]) over a four year period was *not* reflected in test scores. This finding suggests that this IIV estimate may explain unique variances in impairment or decline, as the level of IIV was different for different items on the same test depending on the diagnostic category of the participants being analyzed (normal for four years; normal at first, then diagnosed with AD; or diagnosed with AD from the first visit). This finding further suggests that performance variability as assessed by IIV estimates across items, tests, and/or domains on commonly utilized cognitive tests, might be useful markers of cognitive decline. That is, variability in performance *across items* of a single neuropsychological test may provide a reliable estimate of compromised neural integrity, similar to IIV estimates derived from performance-related measures (e.g., attentional tasks; [25]). This within-test, across-item approach to summarizing intraindividual test performance might have clinical utility, since it may be used to compute estimates of IIV for virtually any cognitive test irrespective of whether or not it comprises reliable subtests; further, estimating IIV would not be limited by the population within which factor analyses to identify those subtests are conducted (e.g., for MMSE-subscore based estimates).

If item-level IIV is useful as a proxy for neural integrity, then it should also predict cognitive decline and impairment similar to the prediction by the total score (sum of the item scores, usually 0 or 1); to our knowledge, no study has reported item-level IIV. However, there are multiple methods for computing IIV. The purpose of the present study was to explore a variety of definitions and compare their effect sizes in order to determine which, if any, could be a clinically useful summary of performance. Using data from the Alzheimer's Disease Neuroimaging Initiative (ADNI), we estimated effect sizes for three commonly-used definitions of intraindividual variability (IIV), namely, intraindividual standard deviation (i.e., computed standard deviation for items or for standardized test scores per person), coefficient of variation (i.e., standard deviation divided by mean, either over all items or over standardized test scores), and level-independent variation (i.e., variability independent of the individual's predicted mean score). We then compared these effect sizes to that derived for the standard, total score-based approach to summarizing performance on the MMSE and Clock Drawing Test-copy. These tests were selected because they are commonly used in both research and practice, and because they contain items that assess global cognitive function based on assessment of various cognitive domains, including language, memory, visuospatial, and executive functions (MMSE: [24]; Clock Drawing: [27]). We further analyzed a single test with specific subscores, the Alzheimer's Disease Assessment Scale – Cognitive (ADAS-Cog, [28]), to compare results across these three different tests.

The primary purpose of this study was to estimate effect sizes (ES) for different IIV definitions and to compare these values to identify the most robust definition of (formula for) IIV derived from cognitive tests, as well as to the ES for either tests' total score. The secondary purpose was to determine whether any of the definitions of IIV were able to differentiate groups with three different levels of disease burden that serve as proxies for structural brain integrity – normal elderly, individuals with mild cognitive impairment (MCI) and individuals with Alzheimer's disease (AD). We hypothesized that IIV would be an informative alternative performance summary (as compared to the total test scores), irrespective of whether it was derived from the items within a single test or derived from multiple tests; because the MMSE is a more ‘general’ test, with items that target a wider variety of cognitive functions than the Clock Drawing Test, we expected that IIV based on MMSE items would yield greater ES estimates than IIV based on Clock Drawing Test items. We also estimated the power of IIV, derived from MMSE, Clock Drawing, and ADAS, to explain variability in change in overall cognitive functioning using a global clinical measure, the Clinical Dementia Rating (CDR, [29]) sum of boxes.

## Methods

### Ethics Statement

Data collection and sharing for this project was made possible by the Alzheimer's Disease Neuroimaging Initiative (ADNI). The ADNI study is IRB approved at all participating institutions (see http://www.adni-info.org/Scientists/ApplyForAccessToSamples.aspx for application process to obtain access to this dataset). Inclusion, exclusion criteria, the list of all sites at which IRB approval was obtained/participant data was collected, and study descriptions are presented at http://www.alzheimers.org/clinicaltrials/fullrec.asp?PrimaryKey=208). The data were obtained de-identified, and were analyzed anonymously.

Our study of the different definitions and formulations of IIV proceeded with simulations based on the MMSE and Clock Drawing Test, as total scores and as the composite of their individual items representing the *context* of variability, with two levels (within- and across-test). Within each context (within MMSE items, within Clock items, and across the 2 tests' total scores) we computed three different *definitions* of IIV (described below). The ADAS is often used as an outcome in clinical trials for treatments in Alzheimer's disease; another clinical outcome for clinical studies and trials is the Clinical Dementia Rating (CDR [29]). Our study of the performance of IIV with respect to the CDR included all three tests.

### Study parameters

Recent investigators [8]–[9] studying across-test IIV formulations on common clinical tests have used the same definition of IIV: the “individual's standard deviation” (ISD), or, the square root of the variance within one person's collected, standardized responses (standardized total scores) on several tests of “cognitive function”. In this study, each individual's standard deviation was derived from the two total test scores (standardized) *as well as* a function of the items on each of these tests singly. A second definition of IIV is the coefficient of variation (CV = standard deviation/mean), derived from both a pair of tests and from the items on the two tests singly. A final definition of IIV was based on an indicator developed by Christensen et al. 2005 [2], termed “mean-independent variation”, MIV, which estimates individual variability but factors out the individual's mean performance. Because it was impossible to recreate MIV specifically with the responses that our data contained (MIV was based on hundreds of trials within multiple blocks), MIV was approximated with a “level” independent measure of variability: each individual's CV was regressed on the total score of the test(s) from which the CV was computed (either the test totals alone or the two standardized test scores together), and the standardized residuals from these regressions represent IIV with the effects of the total score, or level of performance, partialled out. The positive valued standardized residuals (each value was squared, then the square root taken, to eliminate negative values) were used to represent this level-independent estimate of IIV (LIV). When the two total test scores were included in each of these three formulations of IIV, their standardized versions were used and the standardization was based on the mean and variance of the group to which the participants belonged – that is, group scores were standardized depending on the diagnostic cohort individuals came from. The standardized residuals were used to represent this level-independent estimate of IIV (LIV) because the two tests have different scales and distributions. When the two total test scores were included in each of these three formulations of IIV, their standardized versions were used and the standardization was based on the mean and variance of the respective diagnostic groups.

The two factors (definition of IIV, with three levels; context of variability, with two levels (within- and across-test)) yield a 3×2 design for the simulation. Since there were two tests, two different item-level IIV formulations were possible, resulting in a 3×3 design shown in Table 1. The different definitions are outlined in Table 2.

**Table 1:** Designs and definitions for the simulation.

		Context of variability		
		Based on items within a single test		Based on the total scores (standardized) on the 2 tests
**Definition** of intra-individual variability	ISD (individual's standard deviation)	SD for *i*th subject across all items on test 1	SD for *i*th subject across items on test 2	SD for *i*th subject, based on tests 1 & 2 standardized total scores
	CV (coefficient of variation)	SD/mean for *i*th subject, items on test 1	SD/mean for *i*th subject, items on test 2	SD/mean for *i*th subject, based on tests 1 & 2 standardized total scores
	LIV (‘level’-independent variation)	Residual for *i*th subject, CV∼total, test 1	Residual for *i*th subject, CV∼total, test 2	Residual for *i*th subject, CV∼totals, based on tests 1 & 2 standardized total scores

**Table 2:** Definitions and interpretations of IIV formulae.

		Context of variability	
		Based on items within a single test	Based on the total scores (standardized) on the 2 tests
**Definition** of intra-individual variability	ISD (individual's standard deviation) 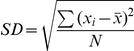	The sum of squared differences between each item's response and the average over all items on the test.	The sum of squared differences between the ISDs for each test ( 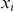 ) and the average over both tests ( 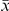 ).
	Like in any distribution, the standard deviation describes how an individual's responses vary relative to their mean. SD is not “corrected” for overall performance. If overall performance limits the amount of variability that can be exhibited (e.g., all right/all wrong will appear not to vary, and be indistinguishable), the SD will not capture that.		
	CV (coefficient of variation) 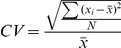	The individual's SD divided by the individual's average over all items on the test ( 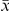 ).	The individual's SD over two tests divided by the individual's average over the two tests ( 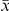 ).
	The coefficient of variation describes how an individual's responses vary relative to their mean, but corrects for the individual's overall performance. This permits comparisons of the variability that remains after accounting for overall performance.		
	LIV (‘level’-independent variation) 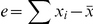	The sum of 1/0 responses on items ( 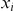 ) minus the average over the responses ( 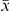 ).	The standardized sums of 1/0 responses on items, standardized for each test ( 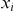 ), minus the average over the responses across all items on both tests ( 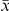 ).
	Whereas CV accounts for overall performance by dividing by it, the LIV type formulation subtracts it. The independence of the variability from the overall performance is estimated as the difference, rather than the quotient.		

### Simulation study design

Clinical data from ADNI (as of October 2008) were used in this study. The baseline (or screening) visit values for items (0 = wrong; 1 = right) and total scores were obtained for individuals participating in the study on two tests; these were chosen because: a) they were recorded in the data files at the item level; and b) they represent a ‘global’ and a more ‘specific’ measure, so that we might observe different results for IIV derived from the items in each. Three types of IIV variables (see Tables 1 and 2) were computed. Table 3 below shows the means and standard deviations for the nine IIV values, plus the two tests' total scores, obtained from the three samples (individuals with a cognitive diagnosis of “normal” (N, i.e., no clinical symptoms AND normal test performance), “mild cognitive impairment” (MCI, i.e., clinical diagnosis based on national criteria) or “Alzheimer's disease” (AD, i.e., clinical diagnosis based on national criteria), based on the baseline visit values for the ADNI cohorts.

**Table 3:** Means and SDs (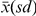) for IIV definitions; these values are the actual sample values from the observed data and were used as “population parameters” for simulations.

	NORMAL	MCI	AD
Total score MMSE	29.11 (0.998)	27.01 (1.789)	23.28 (2.037)
Total score, Clock	9.50 (1.149)	8.81 (1.430)	7.71 (2.025)
ISD MMSE items	0.1246 (0.1143)	0.2784 (0.1078)	0.4159 (0.0465)
ISD Clock items	0.1037 (0.1806)	0.2257 (0.2077)	0.3405 (0.0465)
ISD, 2 total scores	13.8704 (0.9553)	12.8757 (1.4109)	11.0091 (1.7321)
CV MMSE items	0.1324 (0.1239)	0.3179 (0.1360)	0.5455 (0.1085)
CV Clock items	0.1275 (0.2353)	0.3082 (0.3267)	0.5637 (0.4756)
CV, 2 total scores	0.7205 (.0726)	0.7215 (0.0901)	0.7193 (0.1481)
LIV MMSE item cv	0.8714 (0.4827)	0.7346 (0.6757)	0.8383 (0.5369)
LIV Clock items cv	0.7239 (0.6850)	0.8979 (0.4350)	0.6314 (0.7706)
LIV, 2 total score cvs	0.5706 (0.8150)	0.6964 (0.7133)	0.7484 (0.6528)

NOTE: ISD and CV values involving total scores were based on standardized totals on the two tests (using diagnostic group-specific means and SDs). LIV was obtained as the square root of the squared standardized residuals from the regression of the CV on the total score of the test (or, on both total scores, for the 3^rd^ LIV value)- LIV values were all positive (H. Christensen, personal communication).

The means and SDs shown in Table 3 are the sample values for our observed data and represent the summaries (totals, or IIV formulations) that were obtained from the original data. These values were used as “population parameters” to seed the simulations. Based on these values (means and SDs), 500 observations were sampled at random from within each of the three ‘populations’ of summaries assumed to follow normal distributions with the specified mean and SD. This created 500 of each type of test summary from the specified distribution. From these 500 “observations” from the specified distribution, we computed the mean and variance to estimate a single effect size based on N = 500 simulated observations. We then replicated that effect size estimation 500 times, in effect creating a sampling distribution of effect sizes representing the specific comparison (normal vs. MCI; MCI vs. AD) as described below. We chose 500 observations because it is a reasonably large value given the size of longitudinal studies of aging around the country and the world; our results must be reasonable (replicable) by other investigators in this domain and large samples might artificially inflate the precision of estimates.

Based on the three diagnostic groups and the 500 samples simulated for each of the summaries shown in Table 3, two effect sizes were computed in 500 replicated sampling simulations to create the effect size sampling distributions for each of the nine IIV formulations: one comparing the N and MCI groups, and one comparing the MCI and AD groups. The outcome of interest in the simulations was an effect size (Cohen's *d* [30]) derived from each simulated replication (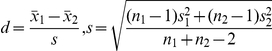) [31], with simulated-sample means and variances and the common group size of n = 500). The effect sizes for AD vs N groups were not evaluated because these two populations are usually easily distinguished. The difficulty, and where the concept of IIV could be most important in future research, is in differentiating the most difficult-to-distinguish groups, which are the ones in adjacent categories, so ES estimates for adjacent diagnostic categories were deemed most interesting for the simulation.

Based on the results of the simulation, we then computed IIV using the single best-supported IIV formula for the observed responses on the MMSE and Clock Drawing Test; we added a third test, the Alzheimer's Disease Assessment Scale-Cognitive (ADAS-Cog [28]) to this analysis, because it is the only one of the three specifically designed for this disease population. We computed multiple regressions (using SPSS v. 17.x, SPSS, Inc. Chicago, Ill) to estimated the power of IIV to explain variability in overall cognitive functioning at baseline, as well as in change in cognitive functioning, using a global clinical measure, the Clinical Dementia Rating (CDR [29]) sum of box scores.

## Results

Table 4 shows the mean of the 500 effect sizes estimated (based on the 500 simulated “observations”) for each of the nine IIV definitions and the two comparisons.

**Table 4:** Effect size estimates for IIV definitions, and total scores.

	NORMAL vs MCI	MCI vs AD
Total score MMSE	1.45	1.95
Total score, Clock	0.530	0.63
ISD MMSE items	1.388	1.660
ISD Clock items	0.630	0.762
ISD 2 totals	0.826	1.182
CV MMSE items	1.426	1.852
CV Clock items	0.939	0.735
CV 2 totals	0.011	0.022
LIV MMSE items	0.232	0.140
LIV Clock items	0	0.426
LIV 2 total scores	0.165	0.077

NOTE: ISD and CV values involving total scores were based on standardized totals on the two tests (using diagnostic group-specific means and SDs). LIV was obtained as the square root of the squared standardized residuals from the regression of the CV on the total score of the test (or, on both total scores, for the 3^rd^ LIV value); LIV values were all positive (H. Christensen, personal communication). IIV estimated based on two total scores were computed using the standardized version of each score.

Table 5 presents the correlations, based on the observed sample data, between the two total scores and the relevant IIV formulations, by diagnostic group. Table 5, which does not represent the simulated data, also shows that ISD versions of IIV are more weakly associated with the respective total test scores than are the CV versions of IIV, although the ISD and CV values based on the combination of the two total test scores (i.e., their combined SD divided by their average) had weaker relationships with the total scores than did the ISD and CV values based on the items. Since higher scores on MMSE, but lower total Clock scores, represent better function, the combination of positive and negative correlations in Table 5 was expected. Table 5 also shows that LIV, computed as positive values (i.e., the square roots of each squared standardized residual values) retained significant relationships with MMSE or Clock total scores; the LIV values for the averaged total scores were not associated with MMSE total score, but LIV values were significantly associated with Clock score in each group. Thus, the LIV formulation of IIV was only partially “level independent.”

**Table 5:** Correlations between *observed* values of the IIV formulations and total scores by diagnostic group from their baseline visits.

NO CLINICAL DIAGNOSIS (N = 229)		
IIV*:	MMSE total	CLOCK/COPY total
ITEMS SD	−.935**	−.900**
TOTALS avg SD	.556**	−.692**
ITEMS CV	−.953**	−.980**
TOTALS avg CV	.042	−.961**
ITEMS LIV	−.337**	−.206**
TOTALS avg LIV	−.057	−.866**

MCI (N = 399)		
IIV*:	MMSE total	CLOCK/COPY total
ITEMS SD	−.934**	−.875**
TOTALS avg SD	.719**	−.501**
ITEMS CV	−.970**	−.983**
TOTALS avg CV	.144**	−.922**
ITEMS LIV	.472**	−.316**
TOTALS avg LIV	.024	−.485**

AD (N = 182)		
IIV	MMSE total	CLOCK/COPY total
ITEMS SD	−.987**	−.740**
TOTALS avg SD	.606**	−.600**
ITEMS CV	−1.0**	−.974**
TOTALS avg CV	.045	−.943**
ITEMS LIV	.441**	−.489**
TOTALS avg LIV	.094	−.375**

*with respect to appropriate test total.

**p<0.0001.

**Figure 1:**
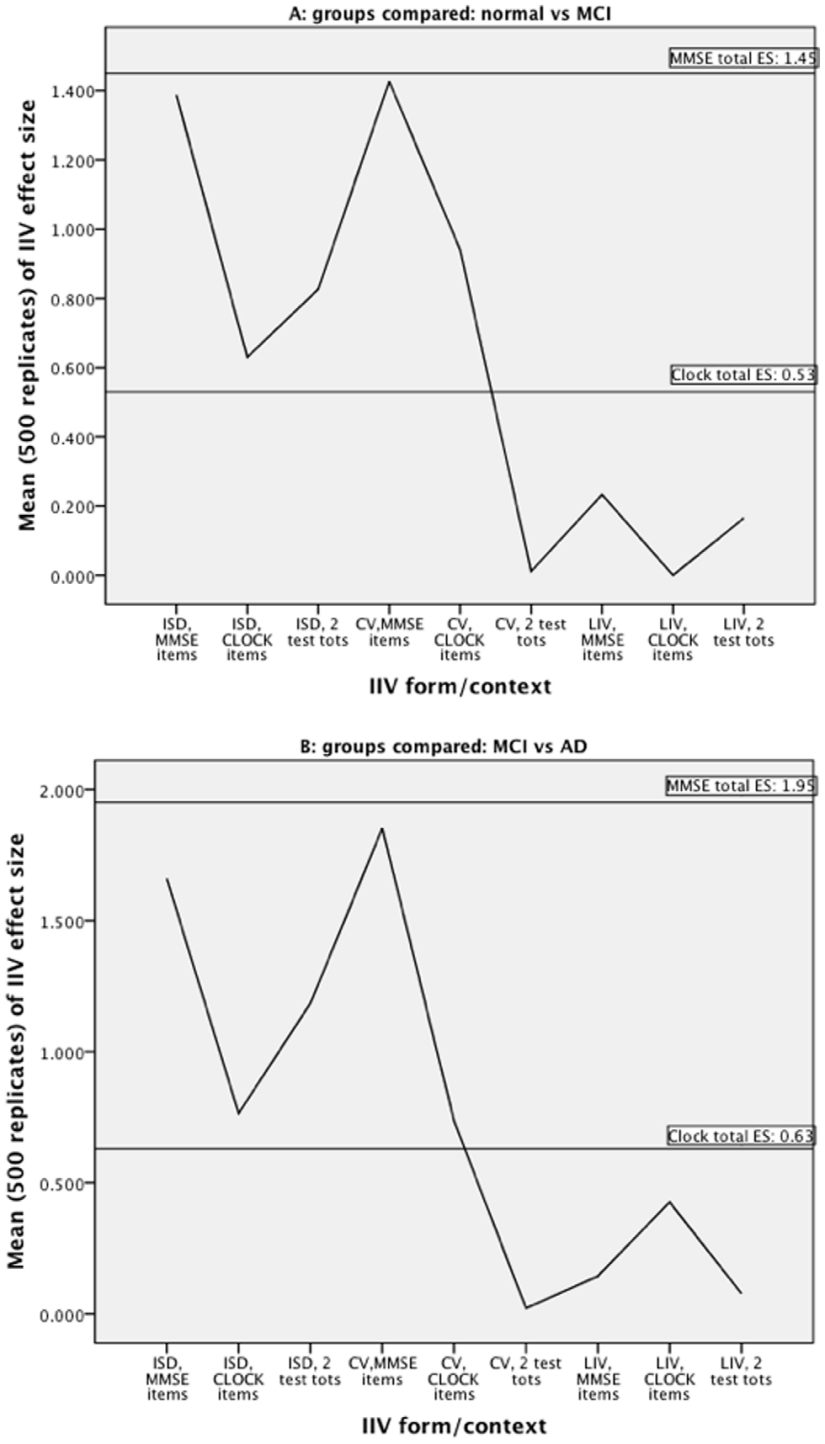
Mean effect sizes based on 500 replications of simulating 500 “observations” for the nine IIV formulations outlined in the text. Reference effect size (ES) values are shown giving the value obtained from the observed data for the total test score (flat lines) (Figure 1A: N vs MCI; Figure 1B: MCI vs AD).

T-tests were carried out to compare the effect sizes estimated for each IIV definition with the ES derived from the total test score(s). Every t-test was statistically significant; thus, in Figures 1A and 1B, wherever one line falls above another, that mean effect size was found to be significantly higher than the corresponding point below it (all unadjusted p values<0.0001).

Visual inspection of Figures 1A and 1B reveals that, for both the N vs MCI and MCI vs AD comparisons, the ES for total MMSE was significantly larger than all IIV formulations, while the ES for total Clock was significantly smaller than the mean ES for most of the IIV formulations. It was observed that IIVs formulated as ISD yielded a stronger ES than CV for Clock items only while CV (mean corrected ISD) had a stronger ES than ISD when based on MMSE items. The figures show that LIV – the formulation of IIV with ‘level’ effects partialled out – produced the weakest ES in all contexts.

Since the CV appears to have generated the most robust effect sizes, we repeated the ES-simulation for baseline values of the ADAS-Cog [20]. Baseline-derived ADAS IIV yielded a smaller ES (N vs MCI: ES = 0.742; MCI vs AD: ES = 0.902) than the total score (N vs MCI: ES = 1.73; MCI vs AD: ES = 1.67); thus the effect sizes for the total score were significantly greater than for IIV in this simulation, and also larger than might be expected generally, just as with the MMSE. Table 6 presents the results of linear regressions to estimate the relative explanatory power of each total score the coefficient of variation (IIV) summary of the same test (individually) at baseline for variability in change over 12 and 24 months in the CDR sum of box scores. In addition to the individual (baseline, BL) summary explanatory power for change in CDR, we estimated the BL IIV summary explanatory beyond that of the total score.

**Table 6:** Statistical explanatory power of within-test IIV (CV) at baseline for change in CDR Sum of Boxes at 12 and 24 months, based on observed data.

Model Results for :		Δ R^2^ for IIV	Δ R^2^ for total	Δ R^2^ IIV, beyond total
To predict change in CDR SB within 12 months(N = 723)	MMSE	0.137***	0.151***	0.001
	ADAS	0.085***	0.214***	0
	Clock Drawing	0.054***	0.044***	0.014**
To predict change in CDR SB within 24 months(N = 594)	MMSE	0.253***	0.280***	0.001
	ADAS	0.139***	0.350***	0
	Clock Drawing	0.142***	0.137***	0.005

** = unadjusted p<0.01;

*** = unadjusted p<0.001; all based on observed data.

Individually, IIV had lower R^2^, relative to that of the total score, to predict change in CDR sum of boxes 12 and 24 months after the item responses were obtained, although IIV itself was a statistically significant predictor of change in CDR sum of boxes scores in all cases. The contributions of within-test IIV (coefficient of variation) above and beyond the explanatory power of the total test scores on the MMSE, Clock Drawing Test, and ADAS-Cog for changes in cognitive symptomatology as reflected by changes in CDR sum of boxes scores was statistically significant for the Clock Drawing Test, which predicted change in CDR sum of boxes over the 12-month interval, but the R^2^ values were very small. Within-test IIV estimates for the MMSE and ADAS-Cog were not associated with change in CDR sum of boxes scores over the 12- or 24-month intervals.

## Discussion

The purpose of this study was to estimate effect sizes (ES) for a set of IIV definitions and to determine which one provided the most robust definition of IIV derived using commonly utilized cognitive tests such as the MMSE and Clock Drawing Test. Results of the study suggested that IIV is an informative alternative performance summary (as compared to the total test scores), irrespective of whether it is derived from the items within a single test or from multiple tests. Consistent with our hypothesis, IIV computations based on MMSE items yielded greater ES estimates than IIV computations based on Clock Drawing Test items. The IIV estimate derived from the Clock Drawing Test predicted cognitive decline above and beyond mean scores on this test at a 12-month follow-up, but other IIV estimates did not.

To our knowledge, this is the first study to estimate effect sizes for a variety of IIV formulations including within test (item-level) estimation using commonly employed cognitive tests such as the MMSE. Our results are not directly comparable to earlier work in the sense that our definitions of IIV varied and we used effect sizes to rank the utilities of these definitions. For example, while “inconsistency” in performance is often estimated using reaction time data and then summarized variability across different tests in terms of the pattern, or dispersion, of test scores (Hilborn et al., 2009 [6]), in contrast, we summarized variability across different tests by computing one estimate of IIV, based on each definition, on combined test performances (see Tables 1A and 1B). In general, our results are consistent with earlier work in that we have shown that IIV, derived from within-test item-level performance or from across test performance, will yield significant effect sizes. That being said, results of this study are not consistent with those of Christensen, et al. [2] who found that a mean-independent estimate of IIV (level-independent variability or LIV) was a useful summary of performance on a reaction-time based measure This may be attributable, at least in part, to the very large number of trials that they used (compared to our study) as well as to the fact that they were using reaction times, not right/wrong answers to questions as we have done here.

Neuropsychological test scores may have a great deal of measurement error (one example is explored in [26]), the constituent items are not exchangeable, and the typical use to which these test scores are put - comparing totals over time to estimate the number of “points lost,” - may have limited interpretive utility. In the context of cognitive science, by contrast, exchangeable and infinitely replicable reaction time trials are perfectly compatible with measuring intra-individual variability (IIV), which may provide useful information regarding the underlying neural integrity of cognitive systems and help predict incident cognitive decline as well as dementia (e.g. [4], [10]–[17], [25]).

Cognitive scientists have suggested that increasing levels of IIV suggest decreasing levels of brain structure/architectural integrity. For IIV to be useful in clinical settings, it should differentiate normal from abnormal cognitive aging (e.g., MCI, AD). Results of this study suggest that IIV *can* be estimated from the items within tests, as well as across cognitive tests, and that the effect size obtained for IIV will depend on the test and on the definition of IIV that is used. Importantly, this study also showed that IIV can be estimated from tests that nearly every NIH-funded Alzheimer's (and clinical cognitive aging) study in the United States is already using, with only the item-level, rather than the total-score level, information.

Methodological limitations of this study must be noted. First, only two tests were used to compute estimates of across-test IIV; this decision was based on the data available and to increase likelihood of replication in future studies. Additionally, the majority of studies on IIV conducted to date have employed reaction-time based tasks. While reaction time tasks may have greater have sensitivity and reliability in measuring IIV compared to the MMSE and the Clock Drawing Test, more research is needed to evaluate this possibility empirically. More research is also needed to identify the number of tests needed to generate reliable estimates of across-test IIV (see, e.g., [32]), and particularly, reliable estimates of clinically meaningful change in variability. Neuropsychological task specificity (e.g., for different brain functions *or* neural circuits, or both) may also need to be evaluated for the best IIV definition for reliable, longitudinal, study of cognitive aging (see also [33]).

A second limitation is that clinical grouping was based on clinical diagnosis. A follow-up study is currently in progress to replicate findings of the current study in neuropathologically-confirmed diagnostic groups. Our results suggest that existing datasets that contain cognitive tests at the item level together with neuropathology and/or neuroimaging outcomes, can be used to explore the hypothesis that IIV can represent, for example, changes in frontal gray matter [13], white matter [14], or neurotransmission [11], [16]–[17].

A third consideration is the many ways to conceptualize effect sizes [30]–[31]; future work will determine the robustness – particularly with respect to longitudinal, clinically meaningful, changes in IIV – to the different effect size estimators. Our analyses have only showed that total scores and the coefficient of variation –computed at baseline- tend to provide similar predictive power for 12- and 24-month changes in CDR sum of boxes; we did not evaluate changes in any of the IIV formulations. It was also unclear why such a large effect size was observed for total score on MMSE, although these larger-than-expected effect sizes were also seen in the MMSE IIV formulations, suggesting the use of MMSE in intake for these subjects might have skewed the MMSE-related results.

A fourth consideration is that we chose to simulate data (generate “random samples”) using the mean and SD of the observed sample as “population parameters” for values following a normal distribution, rather than conduct a bootstrap which would have treated our observed means and variances as if they were the actual population; the bootstrap might have been more supportable if we had exchangeable and infinitely replicable scores. We felt that the more clinical-than-cognitive context of our study and its results supported the simulation approach over the bootstrap approach. It is possible that a bootstrap would have yielded different results, but the simulation is consistent with the way data like ours are used; we will seek to replicate these results in another sample (using simulation) in future work.

Finally, Schmiedek et al. [34] reported that correcting for either individual or group means on a *reaction time*-based estimate of IIV may lead to incorrect inferences. It is unclear whether the same is true for IIV estimated as CV (SD/mean) when the task is not based on reaction times. This is a new, and open, question.

Of interest in Figures 1A and 1B is the unexpectedly large effect sizes of the MMSE total, i.e., the estimated standardized difference (Cohen's *d*, [30]) was 1.45 for the N vs MCI and 1.95 for the MCI vs AD comparisons. These are uncharacteristically large effect sizes, particularly for a general test of cognitive function, in these cohorts. While wholly beyond the scope of this discussion, this particular cognitive test is well known to be very noisy and give very weak effect sizes in general. The IIV-derived ES estimates were also relatively large in several cases. The very large effect sizes documented in the figures above could be due to the use of MMSE in identifying which participants were recruited to the study from which the data were obtained. It is not used to diagnose, but is sometimes used as a shorthand way of referring to –and sometimes recruiting –patients; accordingly, this influence may be driving the dramatic effect sizes that we found. By contrast, the items making up the Clock score were not used to enroll or diagnose ADNI participants. Its total score-based effect size were more modest, .54, for normal vs. MCI and .63 for MCI vs. AD.

A final note is the emphasis in this study on the method of summary, i.e., total (as proscribed) or some version of variability (as shown in Tables 1 and 2). The ADNI study is only one of a large number of similar longitudinal studies presently being conducted. The level of missingness was very low for ADNI data at baseline. Because our results were targeting the simulation, and not so much the original data, we did not address the impact of missingness on our simulations. However, missingness could only have affected the results in Table 6 and would likely have driven our observed-to-be-low estimates further towards zero. These estimates themselves were not the focus of our work but rather, we targeted the difference in using the total score vs. a different summary of the same item level information (i.e., IIV). We did not address missingness or employ random effects models or any kind of imputation in the current study. When IIV formulations and their utility are explored for their use longitudinally, however, missingness and random effects will be important considerations.

Despite these limitations, results of the current study underscore the potential utility of item-level and across-test estimates of IIV in large-scale studies of cognitive aging and dementia. Given that these data are readily available and being collected in longitudinal research protocols, estimates of IIV may provide an additional metric that reflects global neural integrity and may have predictive utility (e.g., [4], [10]–[17], [25]). Definitions of IIV should also be studied for their performance and characteristics longitudinally; in the current study, our simulations and analyses were all based on baseline-data driven IIV estimates. Importantly, although our regression analyses suggested that the total score and within-test coefficient of variation (IIV) at baseline did not provide much explanatory power for change in clinical functioning –and that IIV generally did not provide explanatory power independent of that of the total score, effect sizes for IIV as a summary metric were comparable to ES estimates based on the total scores on these measures. Our future work will focus on studying the performance of IIV estimates longitudinally and developing a better understanding of how variability in response can represent neural integrity and neural pathology.
